# Research Ethical Norms, Guidance and the Internet

**DOI:** 10.1007/s11948-021-00342-5

**Published:** 2021-11-04

**Authors:** Håkan Salwén

**Affiliations:** Department of Philosophy, Stockholm University, Stockholm, Sweden

**Keywords:** Research ethics, Guidelines, Internet, Action-guidance, Tiidenberg

## Abstract

The internet, either as a tool or as an area of research, adds moral worries to an already complicated research ethical backdrop. Agencies, professional associations and philosophers have formulated research ethical norms designed to help scientists to arrive at responsible solutions to the problems. Yet, many criticize this reliance on norms. Somewhat more precisely, many claim that research ethical norms do not offer guidance. In the literature at least three arguments to that effect can be found. First, the research ethical norms fail to guide since they are inconsistent. Second, they fail to guide since they are too opaque. Third, they fail to guide since they cannot handle the moral complexity of issues scientists doing e-research face. In this paper I argue that these arguments are weak. The arguments are, in their original formulations, rather unclear. I try to improve the situation by spelling out the arguments with reference to a certain set of research ethical norms, to a certain account of action-guidance and with reference to certain important distinctions. It then turns out that the arguments’ premises are either untenable or suffers from lack of relevance. The arguments do not force us to conclude that research ethical norms fail to guide.

## Introduction

Researchers that make use of the internet as a tool by using databases and search engines or as a space of research by studying chat forums, homepages or blogs face ethical problems that arguably add to an already complicated research ethical context.^1^ How should researchers respond to problems to assure anonymity in internet-mediated research? Is the scientific value of the project high enough to outweigh failure of full anonymity? How should a researcher establish rapport when she does not know the identities of the participants on chat forums? How should a researcher act on the blurred distinction between private and public? What about the responsibility to give information and gain consent when studying chat forums? Should the information be given even if that affects the reliability of the scientific results negatively and even if the forums are public? (For extensive overviews of ethical issues, see Franzke et al., 2020; Buchanan & Zimmer, 2021).

Governmental agencies, professional associations, scientists and philosophers have proposed research ethical norms designed to help researchers arrive at reasoned answers to questions like these. However, several critics claim that the norms fail to guide researchers that make use of the internet as a space of research. At least three objections to that effect can be found in the literature. The norms fail to guide since they are inconsistent, since they are too opaque and, lastly, since they are not suited to deal with the complexity of the problems that researchers face.

The objections are rather sketchy. Their contents are not spelled out and it is often unclear what their target is. In the research ethical literature, words like ‘principles’, ‘norms’, ‘rules’ and ‘codes’ ‘guidelines’ and the like are frequently used and different authors assign different meanings to them. I will use the expression ‘research ethical norms’ to refer to norms that all scientists are supposed to respect and that are supposed to cover all research practices. In what follows I will evaluate the objections as directed against such norms rather than as directed against the requirements demanded by different ethical review boards relating to, for instance, the precise ways researchers ought to obtain informed consent, how the consent should be signed, documented and filed.

As will be seen in section two, many critics have general norms in mind. Additionally, such norms cover much more than what is covered by review board requirements. This implies that the former have a larger action guiding potential than the latter. Moreover, general norms can be used to defend, critique or adjudicate between particular review board requirements (Bruton, 2014; Resnik, 2018). In consequence, the guidance-objections are more significant as understood as directed against general norms than against specific review board requirements.

General research ethical norms typically include injunctions like: Do not fabricate or falsify data. Treat collaborators with respect. Protect human research subjects from harm. Philosopher David Resnik advocates these and additional norms (Resnik, 2016). He has paid careful attention to them and their relation to research ethical issues in numerous publications and I will formulate his norms in section three. In section four I consider their justification. Action guidance is outlined in section five. In section six I evaluate the strength of the objections. They are essentially philosophical in nature and if we pay more attention to the nature of norms, their justification and make some important distinctions and clarifications we end up with the conclusion that the objections are weak. It is beyond the scope of the present paper to evaluate the objections as directed against the ethical review board requirements. Yet, many considerations stressed in this paper are of significance also to some of the objections as thus characterized.

## Background

Facebook’s well-known emotional contagion study highlights ethical issues e-research gives rise to.

[W]e test whether emotional contagion occurs outside of in-person interaction between individuals by reducing the amount of emotional content in the News Feed. When positive expressions were reduced, people produced fewer positive posts and more negative posts; when negative expressions were reduced, the opposite pattern occurred. These results indicate that emotions expressed by others on Facebook influence our own emotions, constituting experimental evidence for massive-scale contagion via social networks. (Kramer et al., 2014, p. 8788)

The study is scientifically interesting and highly significant to policy makers and to the public. Yet, it is questionable whether the participants had given their informed consent and had the opportunity to opt out from the experiment. Moreover, the participants were manipulated and caused harm.

Some argue that the experiment was morally acceptable and urge scientists to do further Facebook studies in order to learn more about emotional contagion (Meyer, 2014, p. 265). Others oppose this (Recuber, 2016). Should a researcher stick to Meyer’s suggestion or not? Even if it is plausible to assume that research ethical norms can guide researchers in a choice situation like this, the assumption is disputed.

Many claim that research ethical norms do not help scientists doing social media research.^2^Anne Beaulieu and Adolfo Estalella are foremost interested in institutional review board instructions, but do also have more general norms found in ethical guides of traditional disciplines in ethnography in mind. “[C]ertain assumptions embedded in ethics guidelines do not address some of the conditions that are particular to e-research” (Beaulieu & Estalella, 2012, p. 25). Internet researchers, they continue, “have found that some of the principles and categories used in the ethical guides of traditional disciplines are of limited usefulness” (Beaulieu & Estalella, 2012, p. 32). Wyke Stommel and Lynn de Rijk make a similar point. “There have been attempts at creating ethical guidelines for Internet researchers and relevant research ethics committees, [but] no guideline can cover Internet research generally, which leaves a lot of difficult decisions up to the researcher” (Stommel & de Rijk, 2021, p. 2).

Samuel et al. (2019) report, when considering norms for responsible e-research, that scientists as well as members of research ethics committees think that research ethical norms are problematically vague. Samuel, Derrick and van Leeuwen consider are, for instance, the norms issued by The British Educational Research Association, but do not elaborate the relationship between the vagueness and the usefulness of such norms.

In their overview of ethical norms published by Research Councils UK, including guidelines issued by The British Psychological Society and The British Sociological Association, Joanna Taylor and Claudia Pagliari state that their findings “point to a deficit in ethical guidance for research involving data extracted from social media.” (Taylor & Pagliari, 2018, p. 2) Their analysis of the norms “indicates significant gaps in the ethical governance of research using data mined from social media, illustrated by the incompleteness and inconsistency of current guidelines” (ibid. p. 24).

Sociologist Katrin Tiidenberg adds somewhat more philosophical substance to the criticism described above. Tiidenberg targets ethical review board requirements as well as more general guidelines. She claims that the complexity of internet research implies that there are no “clear-cut one-size-fits-all guidelines.” (Tiidenberg, 2018, p. 474). She declares that a number of professional associations have offered such norms but she agrees with Beaulieu’s and Estalella’s complaint that they are of limited usefulness (Tiidenberg, 2020, p. 572). The guidelines are, Tiidenberg continues without elaboration, unable to handle the complex nature of the problems that internet researchers face when it comes to vulnerable groups and sensitive topics. It is,

not possible to create universal, acontextual ethical norms that will ensure the researchers’ ethical behavior. There are often no right answers or good choices; an objective position from which to make ethical judgment does not exist nor can it be explicitly and unambiguously articulated for all to understand. (Tiidenberg, 2020, pp. 573–574)

Tiidenberg’s comment covers a lot of ground. From contested issues within normative ethics about the generality of moral norms, to psychological assumptions about the connection between norms and behaviour, via intriguing meta-ethical issues about the correctness of answers to ethical questions. These comprehensive themes are left without further clarification. Tiidenberg claims, however, that the Belmont principles that uphold US federal regulations, including the ‘Common Rule’ (45 CFR 46, Subpart A), cannot be shaped into workable guidelines. At least, attempts made by ethical review boards end up in guidelines that are “are opaque, inconsistent, and ill-equipped to deal with the complexity of ethical dilemmas encountered by qualitative and internet researchers.” (Tiidenberg, 2020, p. 574).

Tiidenberg and other critics emphasise a number of issues that are highly significant to research ethics. I will consider one important strand in their criticism of research ethical norms, namely that they do not offer guidance for e-researchers. The critics offer at least three reasons. First, the norms fail to guide since they are inconsistent. Second, the norms fail to guide since they are unclear. Third, the norms fail to guide since they are not suited to deal with the complexity of the problems that researchers face, especially those who study vulnerable groups and sensitive topics on the internet. In order to evaluate these objections, they must be related to a particular set of norms or guidelines.

## Research Ethical Norms

Research ethical norms are designed to help scientists to arrive at cogent answers to ethical questions they face in their profession. Supranational organisations like the EU and OECD formulate norms, as do various national agencies and professional associations like World Medical Association and The British Psychological Society. Scientists and philosophers do also formulate and examine research ethical norms.

In what follows I will relate the criticism against the practical significance of research ethical norms all researchers, no matter what scientific discipline they belong to, are supposed to follow. More precisely, I will relate the criticism to norms advocated by philosopher David Resnik. He has published well-known textbooks on research ethics and paid careful attention to research ethical issues in numerous books and articles. Here are Resnik’s norms:

**Honesty** Do not fabricate or falsify data. Honestly report the results of research in papers, presentations, and other forms of scientific communication.

**Openness** Share data, results, methods, and materials with other researchers.

**Carefulness** Keep good records of data, experimental protocols, and other research documents. Take appropriate steps to minimize bias and error. Subject your own work to critical scrutiny and do not overstate the significance of your results. Disclose the information necessary to review your work.

**Freedom** Support freedom of inquiry in the laboratory or research environment. Do not prevent researchers from engaging in scientific investigation and debate.

**Due credit** Allocate credit for scientific work fairly.

**Respect for colleagues** Treat collaborators, students, and other colleagues with respect. Do not discriminate against colleagues or exploit them.

**Respect for human research subjects** Respect the rights and dignity of human research subjects and protect them from harm or exploitation.

**Animal welfare** Protect and promote the welfare of animals used in research.

**Respect for intellectual property** Do not plagiarize or steal intellectual property. Respect copyrights and patents.

**Confidentiality** Maintain the confidentiality of materials that are supposed to be kept confidential, such as articles or grants proposals submitted for peer review, personnel records, and so on.

**Legality** Comply with laws, regulations, and institutional policies that pertain to research.

**Stewardship** Take proper care of research resources, such as biological samples, laboratory equipment, and anthropological sites.

**Competence** Maintain and enhance your competence in your field of study. Take appropriate steps to deal with incompetence in your profession.

**Social responsibility** Conduct research that is likely to benefit society; avoid causing harm to society. Engage in other activities that benefit society. (Resnik, 2016, p. 256–257)

According to one common and important idea, research ethical norms are justified with reference to principles in common morality. These and research ethical norms share important characteristics and the connection between the two is of importance to the evaluation of guidance-objections.

## Moral Justification and Common Morality

Philosophers (see, for instance Resnik, 2001, 2016; Beauchamp & Childress, 2019), and professional associations and governmental agencies subscribe to the idea that research ethical norms are justified with reference to common morality (See, for instance, The Swedish Research Council, 2017, p. 10).

Common morality consists of a number of principles that most people accept. They are simple in character, easy to learn, communicate and practice without deeper analysis. They function as rules of thumb. Common morality includes principles like: It is wrong to steal, you ought to pay your taxes, it is wrong to inflict unnecessary harm, you ought to promote well-being, you ought to obey the law (at least in democracies), you ought to help persons that suffer, it is wrong to punish the innocent, you have right to free speech, promises ought to be kept, you ought to be honest.

Each and every principle pick out a morally significant property. Stealing, unnecessary infliction of harm, and punishing the innocent are all wrong-making characteristics whereas the payment of taxes, promotion of well-being, and promise keeping are right- or ought-making characteristics. The principles are of prima-facie character. This implies that they can all can be justifiably overridden by other norms. Assume that you have promised to visit a friend but when you are on your way you pass a traffic accident involving many persons who suffer badly and are in need of medical care. Suppose that you can easily bring some of them to the nearby hospital. Yet, if you do that you will not be able to fulfil your promise. You face a moral dilemma. One consideration in the situation tells you to take the injured to the hospital, one consideration tells you to go to your friend but you cannot do both. In this case we presumably would argue that the right thing to do is, all told, to take the injured to the hospital. In this case the promise keeping norm is overridden. The obligation to help those who suffer is, in this situation, more important than the obligation to do what you have promised to do. Yet, the fact that this involves the breach of a promise still speaks in favour of the claim that the action is wrong.

Common morality includes a rather heterogenic plethora of principles. Some principles are oriented towards promoting certain ends, others state that some kinds of actions are right (wrong) independent of their consequences and some principles stress various rights. Some philosophers refine these aspects of the principles into full-fledged ethical theories like Utilitarianism, Kantianism and versions of a Theory of rights.

Thus, there is a complex interdependence between different levels in our moral thinking. We have particular moral opinions, we accept various mid-level principles, for instance those formulated in different professional codes and we also subscribe to the norms in common morality. Some also subscribe to ethical theories. These levels are connected and this has important justificatory implications. The more tightly connected the levels are, the more coherent our overall moral system is, and the more justified we are in subscribing to the moral opinions that make up the system (Timmons, 2013). This means that ethical theories are evaluated with reference to their ability to account for the principles in ordinary morality and their ability to account for particular moral opinions. Moreover, principles that fly in the face off our considered moral opinions about certain courses of action are problematic. This means that the rejection of a mid-level principle implies that norms that supported it are problematic and that particular opinions are left without support.

According to one description of the justificatory connection between principles in common morality and research ethical norms, the latter are specifications of the former. Consider for instance the norms to the effect that researchers should not fabricate, falsify or misrepresent data, and that researchers should be open with data, methods and results. These could be said to be a specification of what it takes for a researcher to be honest. Another way to describe the connection is this: We all ought to tell the truth, this is a principle in common morality. Thus, researchers ought to share data since when they do, they are truthful. In a similar way, the common-sense principle that it is wrong to steal justifies the research ethical norm that scientists ought to respect intellectual property and ought not plagiarize since researchers who do not honour intellectual property or who plagiarize do steal.

Notice that just as the principles in common morality are to be understood as qualified by a prima-facie-clause, the research ethical norms must be thus understood. This means that one research ethical norm might suggest that a certain course of action is right whereas another norm suggests that the action is wrong and that one of the norms might be overridden by the other.

So, there is a close connection between common morality and research ethical norms. We need not assume, however, that all research ethical norms are properly described as specifications of common morality principles, or derivable from them. This does not mean that these research ethical norms lack justification. According to the coherentistic model sketched above, research ethical norms might get support from their ability to explain considered moral judgments about particular research ethical cases. Furthermore, many claim that research ethical norms are justified instrumentally. The idea is adherence to the norms advances the goals of science, one of which is to gain knowledge. (Resnik, 2001, p. 68). This idea seems plausible even if it rests on speculative empirical assumptions. Lastly, the norms are intuitively plausible and we do not have any strong reasons to reject them and “[i]t is not irrational to stick to a moral belief when you have no reason to reject it. The fact, if it is a fact, that you lack a positive reason to accept it is not a reason to reject it.” (Bergström, 1996, p. 92).

## Action Guidance

We have already indicated the way in which principles in common morality are action guiding. But, in order to better understand the arguments that research ethical norms fail to guide, we need a somewhat clearer idea what (lack of) guidance amounts to. Here I will describe some important aspects of action guidance. Even if I do not offer an explicit definition of “action-guidance”, the description is for the purposes of the present paper hopefully sufficiently clear.

Philosophers commonly acknowledge that principles in common morality are action guiding (Bales, 1971; Bergström, 1996; Hare, 1981; Timmons, 2013). The norms are helpful in contexts of doubt, they direct our thinking and we make use of them to justify our moral opinions and actions. In choice situations, we often doubt what to do. But when we think about what a certain norm says and apply it to our situation, we might end up with a reason to act in a certain way. At other times we are perhaps convinced that an action is right, but our conviction is challenged. When justifying it, we might claim that the action falls under an acceptable norm.

Let us return to the traffic accident. The norm that one ought to do what one has promised to do as well as the norm that one ought to help those who suffer are action guiding in your choice situation. When thinking of what the first norm says, you get a reason to believe that you ought to visit your friend and a corresponding reason to do so. Yet, this neither implies that you end up with the belief that this is what you ought to do nor that you do it. The reason might be too weak or it might be the case that you have a reason to the contrary. In this case, we assumed, there is a stronger reason to take the injured to hospital. Moreover, the principles are guiding even if we do not know exactly how badly the injured suffer and even if it sometimes is unclear what exactly has been promised.

A norm that you believe is not instantiated in the situation is not action guiding for you in the situation. Suppose that you face a choice situation that does not include references to any promises made. Then, the principle that you ought to do what you have promised to do is not instantiated and thus not action guiding in this situation. Still, the norm is guiding in other choice situations.^3^

The claim that research ethical norms fail to guide scientists doing internet research suggests that for a number of scientists and choice situations, the set of norms does not give the scientists reasons to act in certain ways. What are the arguments for this?

## The Objections

We have described three reasons why the norms fail to guide. They fail since they are inconsistent, since they are opaque and since they are unable to deal with the complexity of the problems that researchers face.

### The Inconsistency Objection

If we understand “inconsistent” in a strict logical sense this objection is false. Resnik’s norms are not inconsistent in this sense. They are all qualified with a prima facie clause, just like the principles in common morality. To the best of my knowledge, no agency, association or philosopher defending research ethical guidelines have denied this.

This suggests another interpretation of the objection, an interpretation in terms of conflict or tension. Norms may “pull” in different directions. The research ethical norms are inconsistent in this sense. For instance, by thinking about what Resnik’s norm about respect of human research subjects says, you might have a reason to think that it is wrong to accept Meyer’s urge to do further Facebook studies. At the same time, by thinking about what Resnik’s norm about social responsibility says, you might have a reason to think that it is right to do so. In many situations where research ethical norms pull in different directions it is difficult to settle what action, all told, is right. Yet, this is not surprising. Ethical problems typically involve conflicts of interests and the identification of stakeholders might be very tricky. Additionally, risk evaluations might be troublesome and long-term consequences difficult to comprehend. In really difficult situations, we might never be in a position to know what actions are right. Still, the norms that apply in a choice situation are action guiding even if they are inconsistent in the sense now assumed. One norm gives us a reason to think that the action is right, another norm gives us a reason to believe that the action is not right. The fact that it might be difficult to know, all told, what is the right thing to do does not alter this. Thus, the inconsistency objection is either false or insignificant depending on the interpretation of the objection.

### The Opaqueness Objection

There are several versions of this objection. According to one version research ethical norms fail to guide since they are phrased in unfamiliar vocabulary. Researchers might simply not understand the formulations of the norms, which therefore fail to guide them. According to another version the norms fail to guide since the norms are too complicated (even if the expressions are understood properly). It might be difficult to settle whether a norm that contains a number of conditions, exceptions or provisos apply in a certain choice situation. However, Resnik’s norms do not have any of these problems. They are phrased in familiar enough vocabulary, without complicating conditions.

But, it might be claimed, it is the vocabulary’s vagueness that makes the norms problematic from an action guiding perspective. This claim is tenuous. The norms are expressed in vague terminology, but this does not show that the norms are unable to guide. In general, vagueness is not a problem when it comes to communication. Consider, for instance, “red”. This expression is vague but it is in most cases not hard to understand or act on injunctions like “Stop at the red light!” or “Pick only red tomatoes!”. The same goes for Resnik’s norms. Even if they are expressed in somewhat vague terminology, it is, due to the very nature of vagueness, in numerous cases clear what constitutes instances of the norms. In these cases, they provide reasons for believing that a certain action is (not) right. The slightly loose character of the norms does not change this. Cass Sunstein, expert on heuristics, claims that: “[I]n most cases they work well despite their simplicity, and if people attempted a more fine-grained assessment of the moral issues involved, they might make more moral mistakes rather than fewer” (Sunstein, 2005, p. 534). Arne Naess argues that certain vagueness is desirable when outlining normative systems (2006, p. 18) and Richard Hare states: “A principle which is going to be useful as a practical guide will have to be unspecific enough to cover a variety of situations all of which have certain salient features in common.” (Hare, 1981, p. 36) Vagueness is, when it comes to guidance, a feature, not a bug.

Those who still raise the vagueness objection take on a heavy argumentative burden. (1) If research ethical norms fail to guide due to their vagueness, then norms in common morality fail to guide due to their vagueness. (2) The norms in common morality do not fail to guide due to their vagueness. So, research ethical norms do not fail to guide due to their vagueness. This argument is logically valid. Those who reject the conclusion must therefore reject at least one of the premises.

What about (1)? Research ethical norms as well as norms in common morality are expressed by ordinary language terms and are thus vague to approximately the same extent. It must then be shown that there is something about research ethical norms that demand a higher degree of precision than that of principles of common morality. There *might* be such a difference, but critics do not explain what it is.

Resnik hints at a possible difference. He claims that honesty is more demanding in science than in ordinary life (2016, p. 256). Still, this does not imply that ‘honesty’ as it figures in expressions of research ethical norms is in need of more precision than when it figures in expressions of norms in common morality. The demand is presumably moral rather than semantic in nature. In scientific contexts honesty might be of more importance than in ordinary life situations, due to trust in scientific methods and results, to knowledge accumulation and to scientists’ status in society,

It could be argued that the demand for precision is higher when it comes to research ethical norms than norms in common morality since there are legal sanctions associated with violations of the former. This argument is not convincing. First, there are not legal sanctions associated with every violation of Resnik’s norms. Second, there are legal sanctions associated with a number of violations of norms in common morality, like murder or theft. So, the alleged “legal” difference cannot be used as an argument for higher degree of precision in research ethical norms.

What about (2)? According to the account of action guidance outlined in this paper, principles in ordinary morality do guide. The rejection of (2) would force us to make major revisions to our moral outlook. It might also force psychologists, cognitive scientists, and philosophers to give up profound beliefs about behaviour in contexts of doubt, about the connection between moral opinions and motivation, and about the very nature of moral opinions. Thus, in order to reject (2) a very powerful argument would be needed. No such argument is made by Tiidenberg or other critics.

We must admit, however, that a researcher might face a choice situation in which it is, due to vagueness, unclear whether one of Resnik’s norms are instantiated. This norm is, for this researcher, in this situation, not action guiding. Yet, there are presumably other norms the researcher believes are instantiated in the situation that offer guidance. Accordingly, even if a particular norm in a particular situation fails to guide due to vagueness, the set of research ethical norms presumably provide guidance in that situation. All in all, research ethical norms are somewhat vague, but this does not mean that they fail to guide.

### The Complexity Objection

The objection is that that the norms fail to guide since they are “ill-equipped to deal with the complexity of ethical dilemmas encountered by qualitative and internet researchers.” (Tiidenberg, 2020, p. 574) This can be interpretated in various ways.

According to one interpretation the objection is that the norms, in a number of cases, indicate or give wrong answers. But what cases and what reasons there are to believe that the answers are wrong? Tiidenberg does not explain.

The objection is, according to another interpretation, that the complexity of the choice situations is such that the norms, as applied to many such situations, do not give us knowledge about what is the right thing to do. This might be true, but it does not follow from this that the norms fail to guide in the sense here presupposed. Compare with the common situation in science where there is empirical evidence for as well as against competing hypotheses. Or compare with theory choice in science. Some considerations, simplicity and fruitfulness, say, speak in favour of one theory, whereas another theory fares better when it comes to generality and explanatory power. These considerations are all of significance. Lack of an algorithm that determines the relative weights of the considerations or lack of a lexical ordering of them do not alter this. Similarly, even if the research ethical norms fail to give us knowledge of what is right to do, they might still give us reasons to believe which action is right and a corresponding reason to perform that act.

The objection might instead be understood to claim that the research ethical norms do not cover a substantive number of choice situations internet researchers find themselves in (Compare with Taylor & Pagliari, 2018). The objection would be that the ethical problems internet researchers face reveal morally relevant properties different from the ones singled out by the existing norms (different in a sense that stealing is a morally relevant property that differs from, say, lying). This implies that there are morally relevant properties that Resnik’s norms fail to cover. Additional norms would then be needed in order guide in choice situations where these properties obtain.

Many internet researchers relate complexity to ways in which informed consent is given, interviews conducted, rapport established, to the ways in which informants’ anonymity and safety are protected and how debriefing is given (see, for instance, Bechmann and Kim, 2020; Ess & Hård af Segerstad, 2019). Yet, this description of the complexity does not, in itself, provide any reason to believe that internet research exemplifies or indicates new morally relevant properties.

There are reasons, though, to believe that there are new ways to comply with or to breach the existing norms. For instance, Tiidenberg’s elaborate description of her ethnographic research with a community of people who post (semi)naked selfies of their bodies online suggests that the internet gives rise to additional ways for researchers to harm, to be open, to (dis)respect human research subjects and to take social responsibility. Yet, this means that the old norms are in play. This is also acknowledged by many agencies and associations (For instance, The Norwegian National Research Ethics Committees 2019 and the British Psychological Society, 2019). The fact that there are new ways to behave (un)ethically might make it very hard to know what action that is right, but, again, this does not imply that the norms fail to guide.

Even so, it might be claimed that the new epistemic setting, induced by the use of the internet as a tool or space of research, is such that researchers in choice situations are undecided whether a certain norm is instantiated in the situations. Let us assume that this is not due to vagueness. For instance, there might be as strong reasons to believe that a certain action is socially responsible as to believe that it is not. Resnik’s norm that scientists ought to be socially responsible is therefore not guiding for these researchers in these situations.

The extent according to which this hold is an empirical question, but my conjecture is that this is a rather rare phenomenon. Also, remember that it is the set of research ethical norms that is supposed to fail to guide in a certain choice situation. It is incredible that the other research ethical norms fail to guide in that situation due to corresponding epistemic stalemates. All in all, the complexity objection fails. It is either left unsupported but significant or tenable but insignificant depending on what version of the objection we consider.

## Concluding Remarks

Scientists often face thorny ethical problems in their profession and the workings of the internet sometimes make the problems even trickier. Most scientists are eager to respond to the problems in a morally responsible way and ask for guidance. Legal frameworks and review board requirements are important but do not settle what the morally reasonable solutions are. The scientist who faces a moral problem in her profession and seeks ethical guidance must therefore resort to another evaluative point of departure, like a set of research ethical norms.

I have considered three arguments to the effect that such norms fail to guide. The arguments, that relate to fundamental philosophical topics (e.g. the nature of norms, epistemic justification, action guidance, inconsistency, vagueness), are as originally expressed too unclear to be evaluated properly. Yet, when formulated in more precise ways, with reference to a certain set of research ethical norms, to a certain account of action guidance and to important distinctions, it turns out that the arguments’ premises are either untenable or insignificant. The premises do not establish that research ethical norms fail to guide. On the contrary, the examination of the arguments gives us reason to believe that research ethical norms do guide researchers in numerous choice-situations.

## Footnotes

1 In what follows I will use “ethics” and “morals” and their derivatives interchangeably.

2 In general, the extent according to which research ethical norms influence scientists’ beliefs about good research practice is unclear. For instance, scientists understanding of scientific misconduct does not seem to relate to severe deviations from such norms (Davies, 2019).

3 The claim that a norm (or ethical theory) is action guiding can be explicated in various ways (See, for instance Carlson 2002 and Smith 2018). The idea indicated above is well in line with a definition that Simon Rosenqvist has recently elaborated in relation to the common objection against utilitarianism that it fails to guide (Rosenqvist 2020). His idea is, in outline, that a moral theory is “guiding because we can use it to obtain reason to believe that an act is right according to the theory.” (Rosenqvist 2020, p. 33).
